# The Effect of a Short-Term and Long-Term Whole-Body Vibration in Healthy Men upon the Postural Stability

**DOI:** 10.1371/journal.pone.0088295

**Published:** 2014-02-10

**Authors:** Magdalena Piecha, Grzegorz Juras, Piotr Król, Grzegorz Sobota, Anna Polak, Bogdan Bacik

**Affiliations:** 1 Department of Physiotherapy Basics, Jerzy Kukuczka Academy of Physical Education, Katowice, Poland; 2 Department of Human Motor Behavior, Jerzy Kukuczka Academy of Physical Education, Katowice, Poland; University of South Australia, Australia

## Abstract

The study aimed to establish the short-term and long-term effects of whole-body vibration on postural stability. The sample consisted of 28 male subjects randomly allocated to four comparative groups, three of which exercised on a vibration platform with parameters set individually for the groups. The stabilographic signal was recorded before the test commenced, after a single session of whole-body vibration, immediately after the last set of exercises of the 4-week whole-body vibration training, and one week after the training ended. The subjects were exposed to vibrations 3 times a week for 4 weeks. Long-term vibration training significantly shortened the rambling and trembling paths in the frontal plane. The path lengths were significantly reduced in the frontal plane one week after the training end date. Most changes in the values of the center of pressure (COP) path lengths in the sagittal and frontal plane were statistically insignificant. We concluded that long-term vibration training improves the postural stability of young healthy individuals in the frontal plane.

## Introduction

The interest in using mechanical vibrations in physiotherapy and sports has clearly grown in the last several years. Although many authors state that vibration training has a positive effect on the human body [1], [2], some published studies have not demonstrated a positive effect [3], [4]. According to Gyulai et al. [5], the reaction to whole-body vibration is training and individual dependent. Whole-body vibration training is an interesting area of research because of its effect on postural stability, and postural stability certainly plays an important role in daily life.

Stabilography is a typical procedure that assesses postural control. Postural stability during static conditions is estimated by analyzing center of pressure (COP) movements in the frontal and sagittal planes, which are recorded with a subject standing in a free standing position on the posturographic platform [6]. The center of pressure measurements are usually reported in postural studies. Zatsiorsky and Duarte [7], [8] have reported a method of decomposing stabilograms. The authors have described the movements of the reference point against which postural stability is instantaneously restored (rambling) and COP oscillations around the reference point trajectory (trembling). The computation method that Zatsiorsky and Duarte [7] have proposed increases the efficiency of investigating the postural control mechanisms and addresses more explicitly the dynamic nature of COP.

The knowledge of vibration effects on postural stability is still unsatisfactory, and available studies present conflicting results. Researchers apply different vibration parameters [frequency and amplitude] and vibration training procedures. For instance, while Polonova and Hlavacka [9] utilized 40–100 Hz vibration, Gomez et al. [10] selected 85 Hz.

Existing studies on postural control systems disturbed by vibration use one of two devices: a vibration platform indirectly producing whole-body vibration [2], [11] or oscillating heads applied directly to the belly of a muscle or a tendon [9]. According to previous research, the effects of vibration are determined by the length of training [11], [12]. A short-term application of vibration to single groups of muscles increases sway [12]. The range and direction of the COP sway depend on the frequency of vibration applied and where the stimulated muscles are located [9]. In the available studies, multiple application of whole-body vibration has either improved the ability to maintain postural balance [13], which indicates improved neuromuscular control [14], or has not changed it significantly [2], [11], [15].

Because studies dealing with the effect of mechanical vibration on human postural stability have yielded diverse results and vibration training methodology has not been standardized, there is a need to conduct more comprehensive research in this field. None of the available publications have used rambling-trembling analysis to study vibration-induced changes in postural stability.

According to Zatsiorsky and Duarte [7], [8] supraspinal processes that are involved in controlling the displacement of the instant equilibrium point may be related to the rambling trajectory, whereas trembling is a reflection of the reflexes and mechanical properties of the muscles and joints. Mechanical oscillations are known to affect kinesthetic perception and produce reflex muscle contractions known as a tonic vibration reflex. Additionally, vibration masks short-latency phasic spinal reflexes by increasing presynaptic inhibition [16]. This allows expecting effects on both rambling and trembling after vibration training.

Therefore, the main purpose of the current study was to establish the effect of whole-body vibration training utilizing different vibration parameters on the postural stability of young males. To analyze the stabilogram, the rambling-trembling approach was applied.

## Materials and Methods

### Subjects

Twenty eight male subjects (22.4±2.4 years) participated voluntarily in the experiment. The mean body mass was 76.3±7.3 kg and the mean height was 179.63±5.1 cm. Young healthy adults that were not professional athletes were recruited. The subjects were randomly allocated to one of four groups; each group consisted of seven subjects. Subjects in groups I-III participated in 4-week whole-body vibration training on a vibration platform, and the frequency and amplitude of vibration were set individually for each group, i.e. 2 mm/20 Hz for group I, 2 mm/40 Hz for group II, and 2 mm/60 Hz for group III. Vibrations below 20 Hz are not used in vibration training studies because it induces mechanical resonance and has an adverse effect on internal organs [17].

A control group (group IV) also participated. These subjects performed exercises similar to those in the other groups but without the concurrent application of vibration.

All participants gave their written informed consent to participate in this study prior to the experiment, which was approved by an ethics committee of the Institutional Review Board.

### Vibration Training

**Figure 1:**
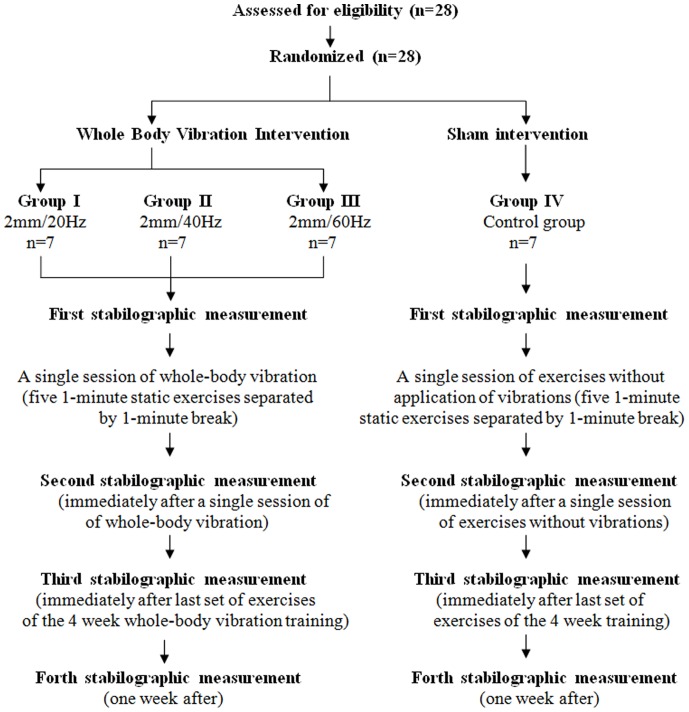
Flow diagram for study design.

The goal of this research was to investigate the impact of whole-body vibration on the stability of the human body. The influence of a single session of whole-body vibration and 4-week whole-body vibration training were assessed. A single vibration session in the study was a series of 5 static exercises on a vibration platform for 1 min, followed by a 1 min break. The study design is presented in Figure 1.

**Figure 2:**
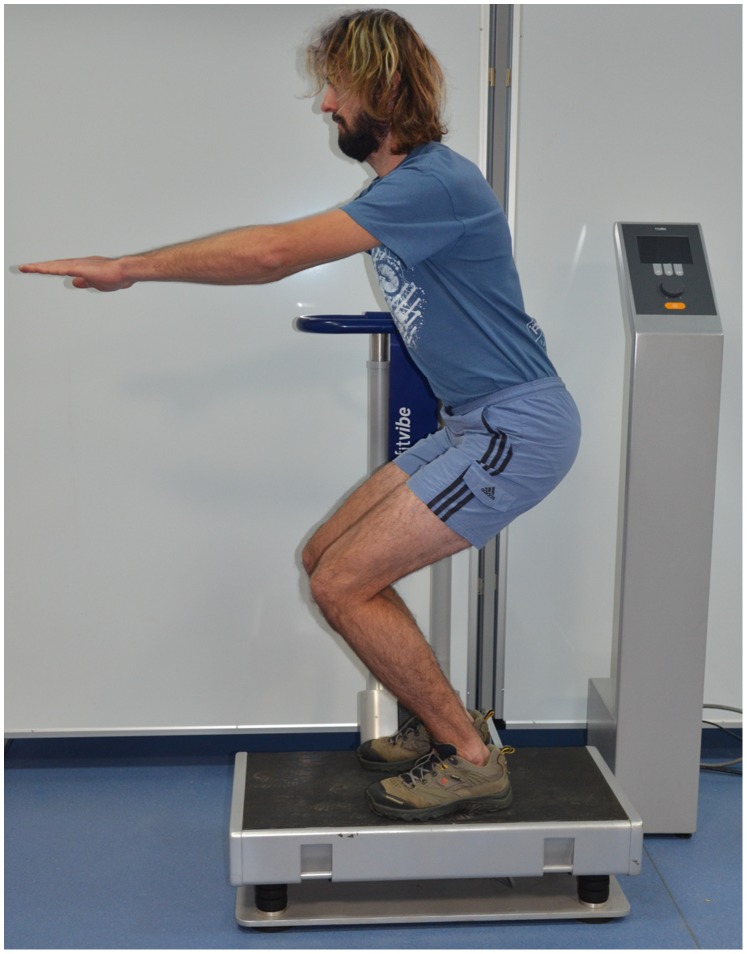
Whole-body vibration.

Whole-body vibration training was carried out on a vibration platform (Fitvibe 600, Gymna Uniphy N.V.). During the experiment, the vibration groups performed 5 one-minute exercises separated by one-minute breaks, 3 times a week (Monday, Wednesday, and Friday). The exercise and rest times were adapted from the procedures used by other researchers [17]. The test participant was in a static position during the exercises. Briefly, each subject was asked to stand on the platform, loading his feet uniformly, with the knee and femoral joints bent at 90 degrees and the upper extremities stretched horizontally forwards (fig. 2). The range of flexion of the hip and knee joints was measured with a goniometer. Krol et al. [18] reported significantly increased bioelectrical activity of the lower extremity muscles of subjects standing with the knee joints bent at 90°. This position is also safer because the knee flexion reduces the amount of vibration that reaches the head.

### Stabilographic Measurement

For the stabilographic measurements, all subjects were asked to maintain a stable neutral comfortable position with their feet shoulders’ width apart, arms lying at the side and eyes closed. Five 30-second trials were recorded on four different occasions: before training started, after a single session of whole-body vibration, after the fourth week of whole-body vibration training, and one week after training ended. Hence, human postural stability was evaluated on 4 different occasions. The postural stability of subjects in the control group was measured in the same manner.

Forces were recorded with a piezoelectric platform (Kistler 9281C) with a charge amplifier (type 9865B) and a computer. Forces were recorded at a frequency of 100 Hz (25 Hz low-pass filter) using the BioWare™ software (type 2812A1-20). The sampling frequency was 100 Hz (25 Hz low-pass filter). The sampling and filtering frequencies were adopted from the procedures of Zatsiorsky and Duarte [7].

All three components of the ground reaction force (Fz - vertical, Fy - anterior-posterior and Fx - lateral components), as well as the three components of moment of force (Mz, My, Mx) about the center of the platform were registered. The COP trajectory was automatically calculated using the Bioware program, and a formula from the guidelines of the manufacturer's platform and its technical parameters was used to determine the position of COP.

The stabilographic signal was obtained using a new method of stabilogram decomposition, proposed by Zatsiorsky and Duarte [7], into two components: rambling (the motion of an instant equilibrium point about which the body’s equilibrium is maintained) and trembling (the oscillation of COP around the reference point trajectory). Using the BioWare™ program, the values of the path length of COP and the path length of rambling and trembling were obtained. The results of Słomka et al. [19] indicate the high reliability of the measures of rambling – trembling.

**Figure 3:**
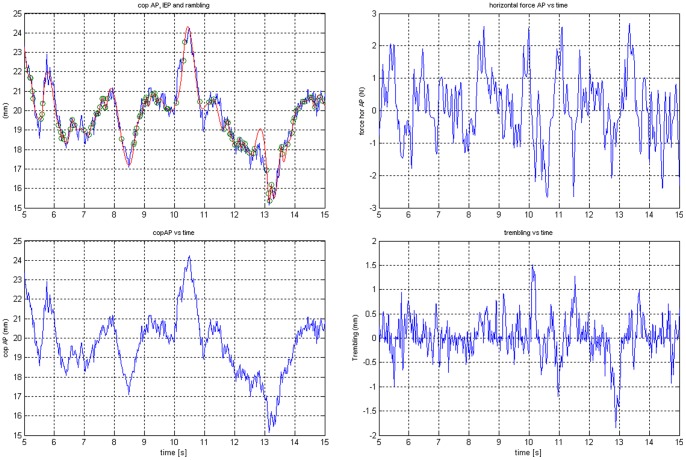
Representative center of pressure, rambling and trembling trajectories of a single subject along the anterior-posterior (AP) axis.

The sequence of operations used for stabilogram decomposition is described below [20] (fig. 3). When the horizontal force (Fhor) is zero, the body is in an equilibrium state. These instances were identified in the COP displacement data. The COP positions when Fhor is 0 were located, and the signal between each point was interpolated using a cubic spline function to obtain an estimate of the rambling trajectory. The COP trajectory is compared with the interpolated rambling trajectory. To obtain the trembling trajectory, the deviation of the COP from the rambling trajectory was calculated.

### Statistical Analysis

The Shapiro-Wilk test was used to check the data for normal distribution, while variance homogeneity was investigated using Levene’s test. Because some parameters failed to meet the assumption regarding the normal distribution of variables and variance homogeneity, the Wilcoxon matched pairs test was employed to assess the significance of the differences between the means of particular variables in the groups.

The influence of the vibration frequencies and amplitudes on the means of particular variables was verified with the Mann Whitney U test. The intergroup statistical analysis used the relative values of the difference variables, which were calculated as the ratio of the difference between the initial and final value of a variable to its initial value:
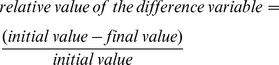



The level of statistical significance for all analyses was considered p<0.05.

## Results

**Figure 4:**
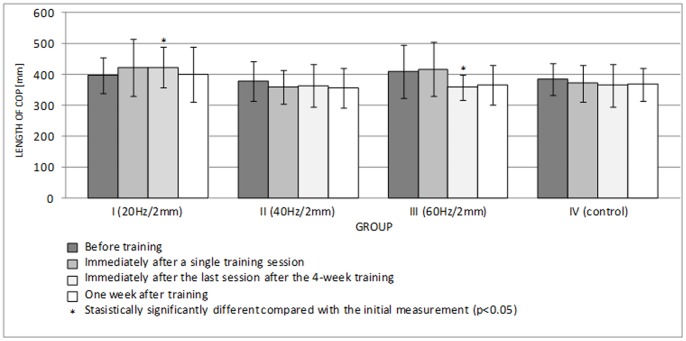
Length of COP in the AP plane (mean ± standard deviation) (mm) after training.

**Figure 5:**
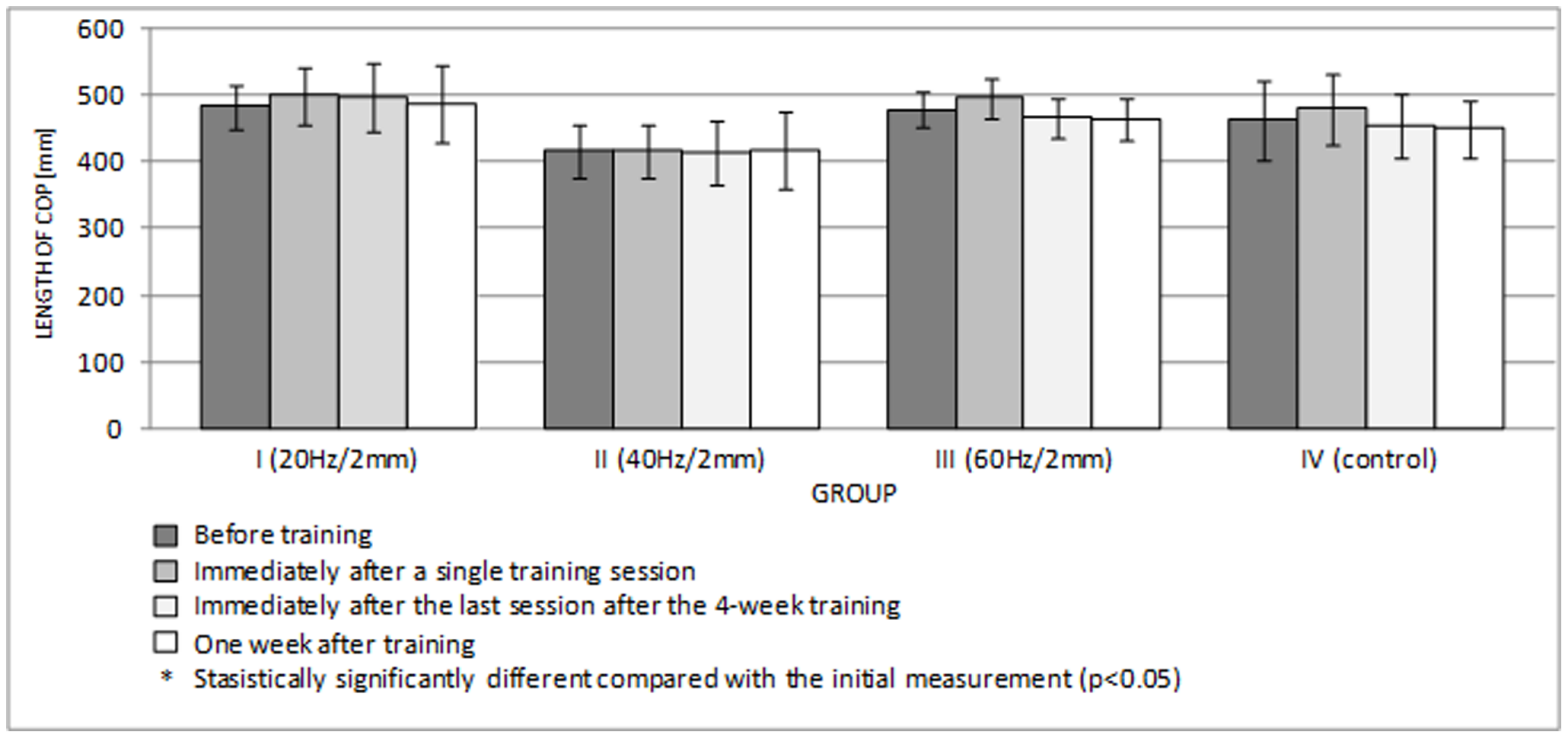
Length of COP in the ML plane (mean ± standard deviation) (mm) after training.

Both a single application of vibration and the 4-week whole-body vibration training had an effect on the COP path length values in the sagittal and frontal planes, but most changes were statistically insignificant (p>0.05) (fig. 4, 5). Group I was the only group where the COP path length in the AP plane was significantly increased after the last session of the 4-week whole-body vibration training (p = 0.046) (fig. 4). In contrast, the path length of COP in group III was significantly decreased after the last session of the 4-week whole-body vibration training (p = 0.027) (fig. 4).

**Figure 6:**
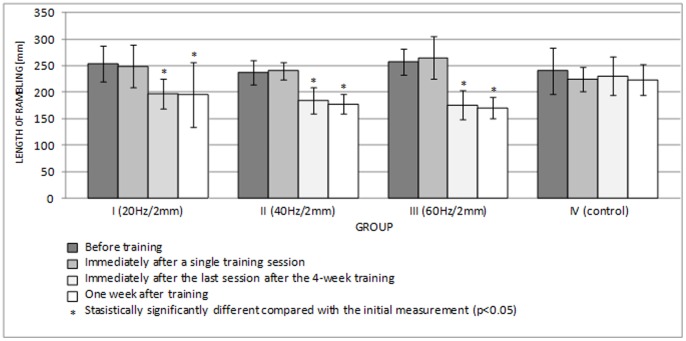
Length of rambling in the AP plane (mean ± standard deviation) (mm) after training.

**Figure 7:**
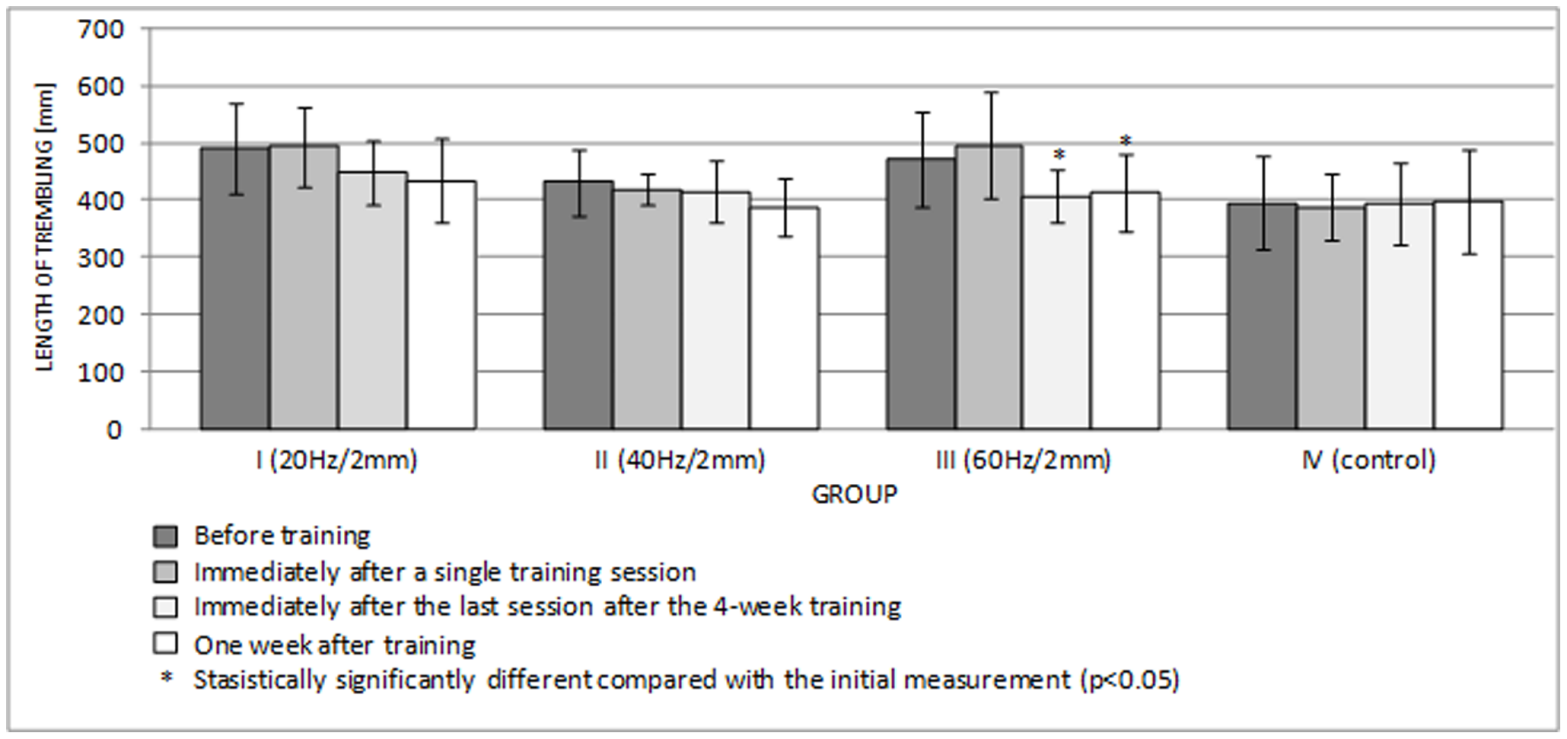
Length of trembling in the AP plane (mean ± standard deviation) (mm) after training.

**Figure 8:**
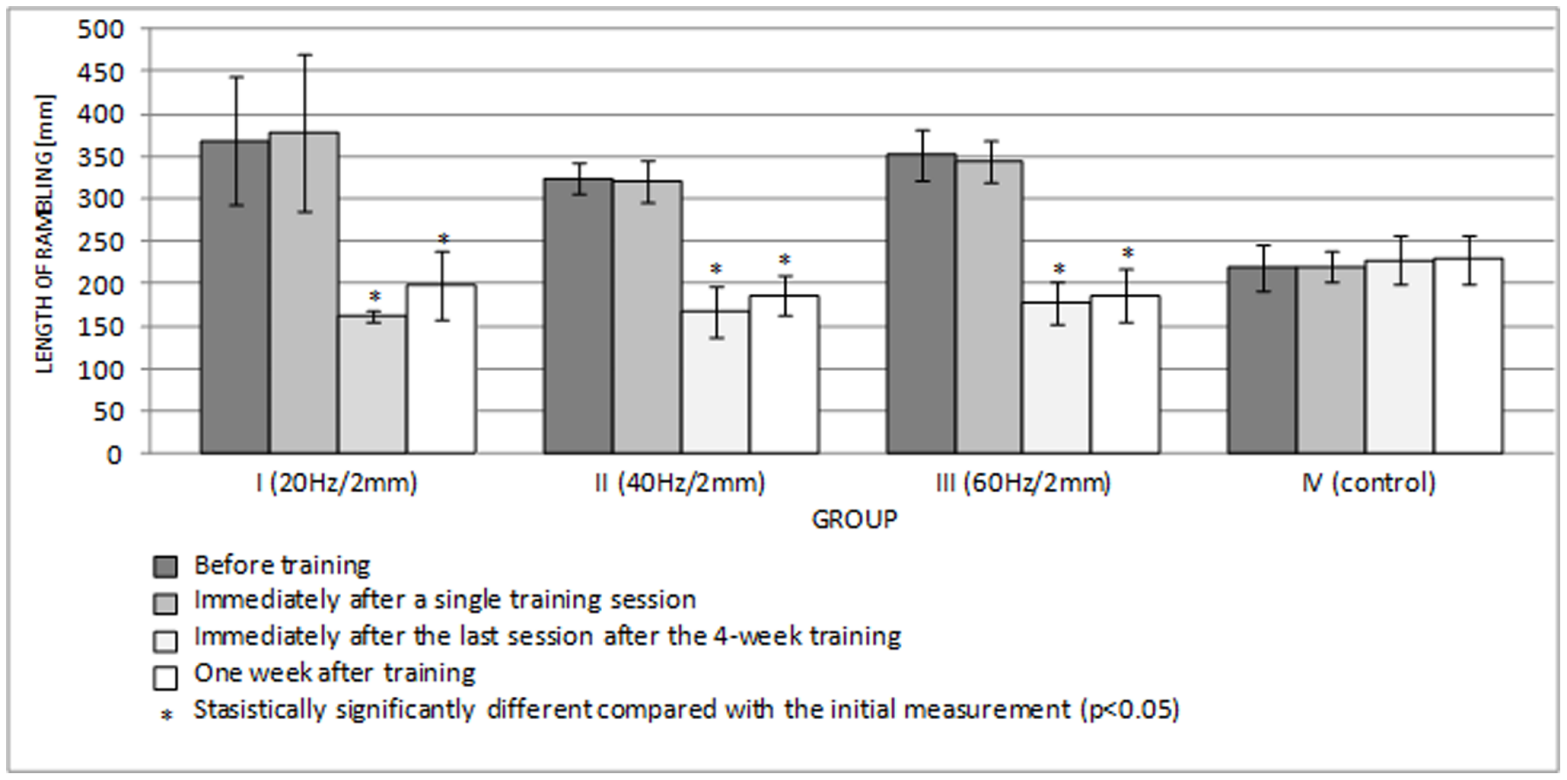
Length of rambling in the ML plane (mean ± standard deviation) (mm) after training.

**Figure 9:**
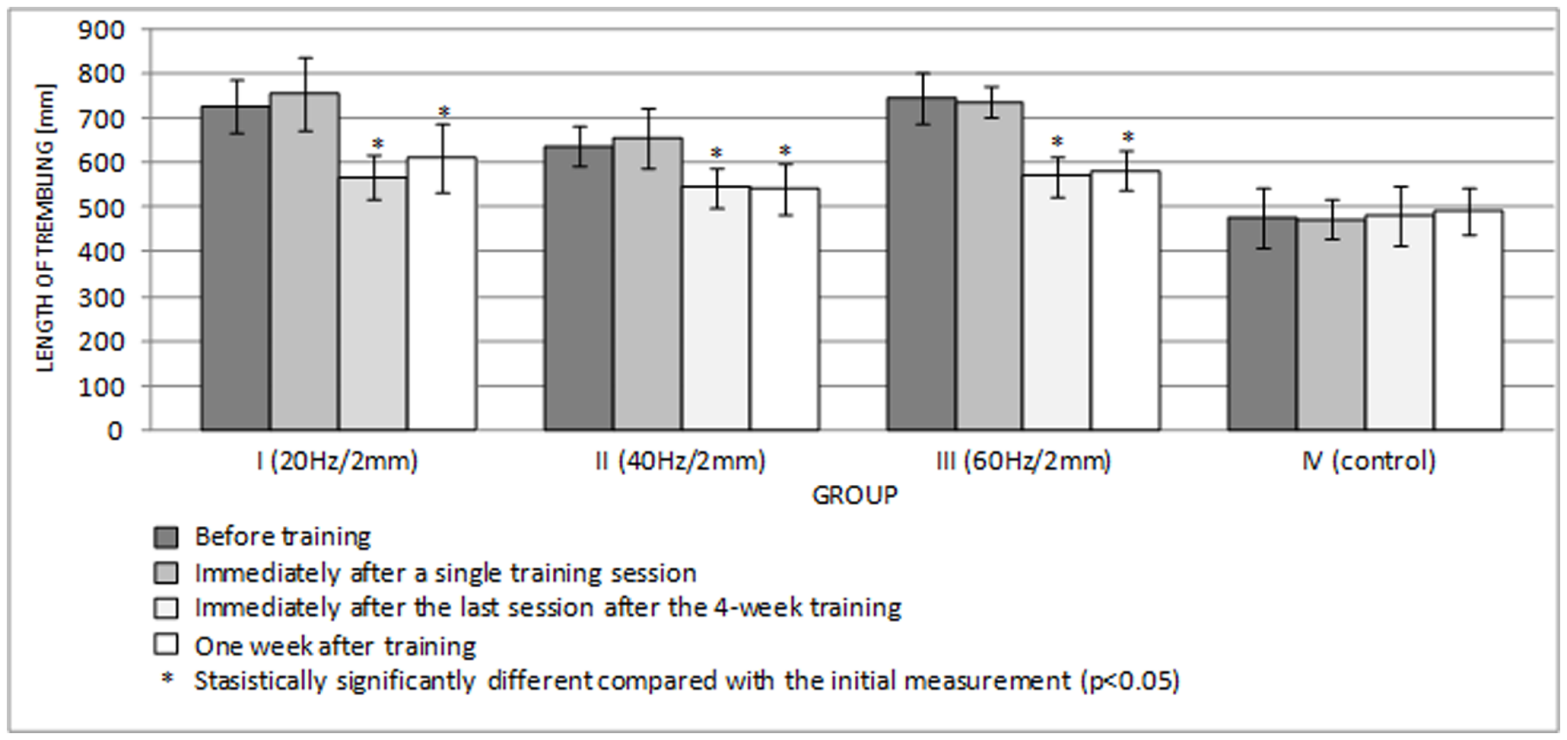
Length of trembling in the ML plane (mean ± standard deviation) (mm) after training.

Figures 6 and 7 show the lengths of the rambling and trembling paths in the sagittal plane, and figures 8 and 9 give the same information for the frontal plane.

A single session of whole-body vibration training did not significantly affect the path lengths of rambling and trembling in the sagittal plane (fig. 6, 7) (p>0.05) or in the frontal plane (fig. 8, 9) (p>0.05). In groups I-III, long-term vibration training significantly shortened the rambling path in the sagittal plane (fig. 6) (p<0.05). In group III, the significantly shorter trembling path in the sagittal plane (p<0.05) after 4 weeks of vibration training is notable (fig. 7).

Interestingly, statistical analysis showed significantly shorter rambling and trembling paths in the frontal plane (p<0.05) (fig. 8, 9) for all test groups participating in 4-week vibration training (excluding the control group). This phenomenon turned out to be relatively long lived; statistical analysis showed that the rambling and trembling paths were significantly shorter in all test groups (excluding the control group) one week after the training end date (p<0.05) (fig. 8, 9).

To establish the impacts of different vibration frequency and amplitude parameters, test groups I-III were compared with each other. The Mann Whitney U test did not show a significant influence of vibration frequency and amplitude on the path length of the COP and rambling and trembling paths (p>0.05) between the pre-test measurements and those made directly after the first session of whole-body vibration, immediately after the last set of exercises of the 4-week whole-body vibration training and one week after.

## Discussion

The complex system of postural control includes a variety of elements: motor synergies, sensory input, anticipatory postural adjustments, and adaptive control [21]. Appropriate postural adjustments, i.e. kinematic and kinetic adjustments, are aimed at keeping the center of gravity (COG) over the base of support [22]. According to Collins and de Luca [23], during quiet standing, the postural control system utilizes open-loop and closed-loop control schemes over short-term and long-term intervals, respectively. All forms of postural activity during normal standing in healthy young males are cyclical in nature and include high and low frequency components [6]. The size of the base of support is an important factor influencing the postural stability [24].

An important aspect of postural control that enables people to move safely in the immediate environment is the processing of information received from four sensory entrances – the labyrinth, the organ of sight, the proprioceptors and the tactile receptors [24]. If sensory receptors receive incorrect information, then postural stability may be affected [25]. Based on the published results, several different types of receptors may be sensitive to mechanical oscillations, for example, extroceptive receptors of the foot (Merkel, Meissner, and Ruffini receptors) [26] and proprioceptors [27]. Primary endings are the most sensitive to vibratory stimulation [28]. Brumagne et al. [27] have demonstrated that local vibration of paraspinal muscles impairs proprioception in healthy humans. In our experiment, proprioceptors were disturbed by mechanical vibrations of different frequencies (20, 40, and 60 Hz) and the same amplitude of 2 mm.

Many studies exploring vibration effects on postural stability have been conducted [2], [11], [12], [13], [15], but their results are diverse and inconsistent. There is still a lack of standardization regarding the testing and training protocols for whole-body vibration, which makes it difficult to compare the various research studies. There are differences in the application methods for vibration. Direct vibration is normally locally applied to a muscle or tendon with oscillating heads at high vibration frequency (100–150 Hz) and small amplitude (1–2 mm) for a short period of time (2–15 s). Indirect vibration typically uses vibration platforms producing whole-body mechanical oscillations with lower vibration frequency (25–45 Hz), higher amplitude (2–10 mm) and longer duration time of either continuous (3–5 min) or intermittent exposure (30–60 s) [29].

In the available studies, multiple application of whole-body vibration training has either improved postural stability [13] or has not resulted in significant changes [2], [11], [15]. The improved postural stability in patients might be due to increased muscle strength, improved synchronization of the firing of motor units and improved co-contraction of synergist muscles after whole-body vibration training [17].

Center of pressure (COP) measurements are commonly used output measures of the postural control system, as they are indicative of postural stability. A short-term application of a direct vibration of 60 Hz and an amplitude of 1 mm to single groups of muscles increases sway [12]. Indirect vibration, which is applied to the whole body with frequencies of 20, 40, and 60 Hz and amplitudes of 2 and 4 mm, does not significantly change the sagittal and frontal COP sways in young males [15]. In our experiment, the path length of COP changed in all test groups, and most changes were statistically insignificant (p>0.05). Additionally, the first set of exercises involving the vibration platform changed the lengths of the rambling and trembling paths in the sagittal and frontal planes in test groups I-III, but the differences were not statistically significant (p>0.05). This lack of effect likely results from compensatory postural adjustments.

According to Winter [30], there is a certain degree of redundancy that can be applied when one or more of the sensory systems fails or is temporarily lost. Healthy humans use compensatory information from the complementary sensory systems (visual, vestibular and proprioceptive) for postural control [31]. The re-ranking of receptor information as a result of disturbed postural stability has been discussed by many authors [32], [33]. Too much sensory information or malfunctioning of any of the receptors causes the nervous system to reduce the receptor’s role in postural control and switches to signals from receptors offering more reliable and noise-free information. The hierarchy of receptors depends, *inter alia*, on the motor task requirements [33] and receptor efficiency [32].

Dolny and Reyes [34] are of the opinion that exposing young, healthy individuals to whole-body vibration may not be sufficient to induce adaptive changes in their neuro-muscular system. A single session of whole-body vibration in our experiment was most likely too weak of a disturbing stimulus to modify postural sway. According to its authors, a possible explanation of why short-term vibration did not significantly differentiate the path length of COP and the lengths of the rambling and trembling paths between the tested groups, should be found for young subjects going through a period of ontogenetic development, with its relative balance between excitation and inhibition processes.

In our experiment, the vibration application time was 1 minute, similar to the studies of other authors [18]. According to Cochrane [35], vibration applied continuously for longer than 1 minute causes muscle fatigue that is known to increase postural instability in the ML and AP directions [36]. As shown by the results of this experiment, five 1-minute static exercises did not induce muscle fatigue in any experimental group.

Zatsiorsky and Duarte [7] have proved that the horizontal force and the trembling component are highly and negatively correlated with each other. Each time the COP deviates from the interpolated IEP path, a horizontal force tending to reduce the deviation is triggered [7]. In the experiment, the moderate amount of deviation from the rambling path suggests that the source of corrective force was the apparent intrinsic stiffness of the lower extremity muscles of the subjects [37]. According to Winter et al. [24], reduced postural sway can be explained in terms of the increased stiffness of the ankle joints primarily caused by the tension of the postural muscles that stabilize them. This corrective force is released instantaneously [7].

Tahayor et al. [38] suggest that during peripheral perturbation, different strategies are involved in posture control. These changes in the dynamics are related to adaptive strategies that aim to control postural sway [38]. Repetitive mechanical stimulation might lead to a rearrangement of postural control strategies [39]. Because the cyclical exposure to whole-body vibration frequently causes adaptive changes in the COP signal [40], we acted on the hypothesis that 4-week whole-body vibration training should induce permanent changes in the amount of postural sway. The statistical analysis results proved that this hypothesis was true. In all test groups participating in the whole-body vibration training, the rambling and trembling paths were significantly decreased in the frontal plane after 4 weeks of exposure vis-à-vis the initial measurements (p<0.05). Interestingly, the difference continued for a long period. Statistical analysis showed that the paths were significantly shorter even one week after the training program ended (p<0.05). The changes deserve special attention because the statistical significance obtained for the small groups (n = 7) clearly shows that the result is valid.

Shorter path lengths in our experiment may signify that regular, long-term vibration training caused permanent adaptive changes in the postural stability of the subjects. According to many authors, vibration training induces adaptive changes in muscle tissue as well [2], [11]. The long-term vibration training used in our experiment most likely affected the viscoelastic stiffness properties of the muscles [41].

Wierzbicka et al. [41] demonstrated long-lasting dynamical changes in postural stability after local vibrations. According to Duclos et al. [42] these long-lasting alterations might originate from sustained artificial activation of the somesthetic mechanoreceptors, which results in modification of the central integration of this sensory information. The activation of sensorimotor cortical networks by proprioceptive signals, induced by mechanical oscillation, remains after the vibratory stimulation is completed [42].

In a human standing with natural posture, the amount of sagittal sway is greater than the amount of frontal sway [24]. Thomas and Whitney [6] found that in healthy, young adults, the average amplitude of anterior-posterior movement of COP is 0.60 cm. Greater sway in the sagittal plane is shown by the results obtained for all test groups in our experiment (fig. 4, 5). It is assumed that in the sagittal plane, the human body behaves similar to a one-piece, inverted pendulum with the rotation axis going through the ankle joints. In discussing upright posture control, Kuczyński [37] refers to stabilization ensured by springs with absorbers to describe the viscoelastic properties of the muscles. Frontal stability is determined by the bipedal stance and substantially restricted the mobility of the joints of the lower extremities in this plane [24]. In the quiet standing position, with the feet side by side, the ankle strategy is primarily applied in the sagittal plane, whereas a separate hip load/unload strategy is the dominant strategy in the frontal plane [30].

Because the COP measurements were not significantly sensitive to vibration training in this experiment, the rambling-trembling technique may be more suitable, as it allows discrimination between supraspinal and spinal processes. Additionally, rambling-trembling parameters have a higher reliability coefficient than COP parameters [19].

This study aimed to establish the short-term and long-term effects of whole-body vibration training on postural stability. As an experimental study, it has known limitations. A small sample size in each group may not be sufficient to establish full training effects. Healthy, young males most likely have very good balance at baseline. Therefore, it seems worthwhile to conduct multifaceted research among older people and patients with postural balance problems. Comprehensive and reliable studies with an innovative use of vibration platform [43] are still required.

### Conclusions

While aware that further studies exploring the influence of various types of whole-body vibration training on postural stability are necessary, current research conclusions suggest the following:

1. Long-term whole-body vibration training significantly shortens the rambling and trembling paths in the frontal plane.
2. Rambling and trembling paths are significantly decreased one week after whole-body vibration training compared with the initial measurements.
3. Rambling – trembling decomposition is a more sensitive method for the analysis of quiet standing than classical stabilometric parameters of COP.
